# Polymeric and Lipid Nanoparticles: Which Applications in Pediatrics?

**DOI:** 10.3390/pharmaceutics13050670

**Published:** 2021-05-07

**Authors:** Noelia Nieto González, Antonella Obinu, Giovanna Rassu, Paolo Giunchedi, Elisabetta Gavini

**Affiliations:** 1 PhD Program in Chemical Science and Technology, Department of Chemistry and Pharmacy, University of Sassari, Via Muroni 23/a, 07100 Sassari, Italy; nnietogonzale@uniss.it; 2 Department of Chemistry and Pharmacy, University of Sassari, Via Muroni 23/a, 07100 Sassari, Italy; aobinu@uniss.it (A.O.); pgiunc@uniss.it (P.G.); eligav@uniss.it (E.G.)

**Keywords:** nanomedicine, polymeric nanoparticle, lipid nanoparticle, pediatric disease, pediatric medicine

## Abstract

This review aims to provide the state of the art on polymeric and lipid nanoparticles, used or suggested to approach pediatric diseases’ problems and needs, and to inspire new researches in this field. Several drugs are currently not available in formulations suitable for pediatric patients. The United States Pediatric Formulation Initiative suggested applying new technologies to pediatric drug formulations, for instance, nanotechnology. The literature analysis showed that polymeric and lipid nanoparticles have been widely studied to treat pediatric diseases, and albumin nanoparticles and liposomes are already used in clinical practice. Nevertheless, these studies are focused almost exclusively on pediatric cancer treatment. Although nanomedicine may solve many needs of pediatric diseases and medicines, the unavailability of data on pharmacokinetics, safety and efficacy of both drugs and nanoparticles in pediatric patients limits the development of new pediatric medicines based on nanoparticles. Therefore, nanomedicine applied in pediatrics remains a significant challenge in the near future.

## 1. Introduction

**Figure 1:**
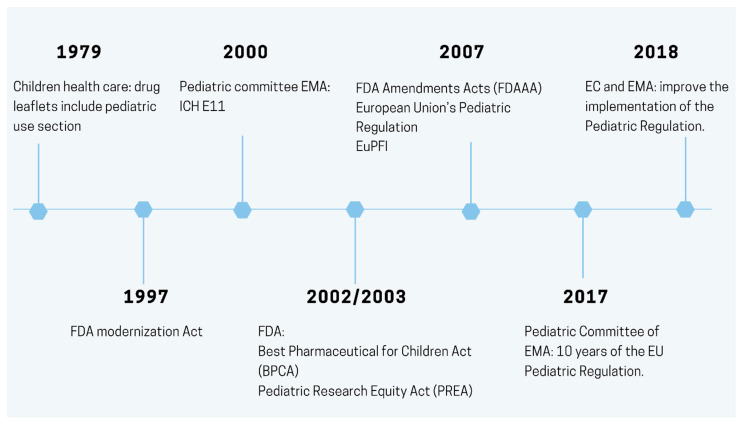
Schematic representation of the key milestones in the regulation of pediatric medicines.

The European Union’s (EU) Paediatric Regulation took effect in 2007. It aimed to encourage research in age-appropriate formulations and to raise high-quality information on pediatric medicines. This Regulation was inspired by United States (US) Pediatric Legislation, whose legislative approaches have been developed since 1997 [1]. Since the 1980s, it has been argued that pediatric patients deserve the same level of health care as the rest of the population [2,3] (Figure 1). The report published in 2017 by the European Commission (EC), on the 10th anniversary of the EU Regulation, shows an increase in children’s medicines. The most significant advances have been made in the treatment of diseases common in adult and pediatric patients.

Nevertheless, in the context of rare or exclusive children’s disease (e.g., some forms of tumor), the impact was minor [4]. However, since the Regulation’s implementation, from 2007 to 2015, 238 new medicines for pediatric patients and 39 new formulations appropriate for this population were authorized in the EU [2]. The Paediatric Committee at the European Medicines Agency (PDCO) has established an inventory that defines the requirements for various pathologies and drug formulations in different age groups in the pediatric population to promote their investigation [5]. The European Paediatric Formulation Initiative (EuPFI) was founded in 2007 to study the different needs related to pediatric formulation development in order to formulate better drugs for children [6]. On 20 March 2018, in a workshop organized by the EC and European Medicines Agency (EMA), one of the topics discussed was again identifying pediatric medical needs [4].

Several drugs are currently not formulated for administration to children. In hospitals, they are frequently formulated in galenic medicines, using adult dosage forms or off-label use. Surveys suggested that in many therapeutic areas, off-label use was higher than 50% [3]. Moreover, the use of extemporaneous preparation may result in an elevated risk of toxicity or low therapeutic dose, an uncertain bioavailability and an absence of documented information about the stability [7].

The development of pediatric medicines is complex, because children cannot be considered small adults and heterogeneous. The EMA classifies them into five groups: (i) preterm newborn infants, (ii) term newborn infants (0–27 days), (iii) infants and toddlers (1 month to 23 months), (iv) children (2–11 years), (v) adolescents (12–16 or 18 years). Each age group has different requirements considering the type of formulation and the administration route due to physiological, anatomical and pharmacokinetic changes [7]. The optimal considerations for pediatric formulations are (i) age-appropriate dosage forms, (ii) facility of preparations and instructions for use for careers, (iii) acceptability, (iv) choice and quantity of excipients, (v) use of alternative delivery systems, and vi) appropriate packaging to improve efficiency and avoid the risk of dosage errors [8]. Also, excipient choice for enhancing drugs is one of the main issues in the pediatric area. Excipients safely and commonly used in formulations for grown-ups could be harmful to children [7]. For example, regarding preservatives, the use of benzyl alcohol should be considered carefully, and it is better to avoid it in young pediatric patients due to their immature metabolism [9]. Also, ethanol shows adverse synergistic effects on the central nervous system if combined with other drugs. For example, ethanol and dextromethorphan can cause an infant’s death [10].

The United States Pediatric Formulation Initiative (US PFI) suggested applying new technologies for pediatric drug formulations. Nanotechnology is considered an innovative approach for reducing drug toxicity and improving drug efficacy. US PFI also argued for future applications of polymeric nanoparticles as a controlled drug delivery system over a long period [11]. This review aims to assess the nanoparticles (NPs) used or suggested as drug delivery systems in the pediatric field to tackle pediatric diseases’ needs. The excipients used and the technological and biological properties of the nanosystems are reported.

## 2. Nanoparticles as Pediatric Formulations

NPs are particles with a size range from one to several hundred nanometers. The active agents are incorporated in the core or on the surface of the particles [12]. They can be used as delivery vehicles of small drugs, diagnostic imaging agents, genes, proteins and peptides. NPs can positively alter the pharmacokinetics of drugs and, if compared to conventional pharmaceutical dosage form, lead to a different dose-response [13]. So, there are challenges in pediatric nanomedicine.

Regarding pharmacokinetics, the drugs formulated in nanosystems displayed a prolonged circulation time, increased half-life, reduced clearance, and increased mean residence time [14]. These improvements were obtained, for example loading doxorubicin, daunorubicin, vincristine and amphotericin B, in liposomes [12,13,14]. NPs show a fundamental role in modifying physicochemical properties, improving water solubility, stability and permeability of the drug [15]. Moreover, NPs provide advantages in pediatrics because they could reduce the dose of antineoplastic drugs and their side effects [16].

**Figure 2:**
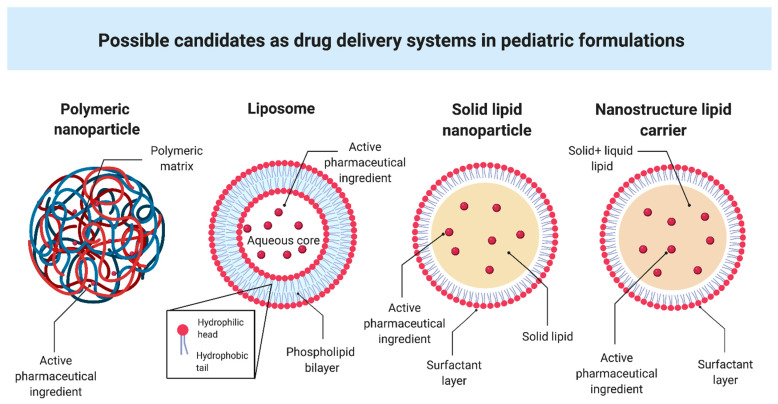
Schematic representation of possible candidates as drug delivery systems in pediatric formulations. It was created with Biorender (https://biorender.com/ accessed on 27 April 2021).

NPs can be classified according to their chemical and physical characteristics into polymeric NPs, lipid-based NPs, metal NPs, ceramic NPs, semiconductor NPs and carbon-based NPs [17]. Polymeric and lipid NPs (Figure 2) are the leading candidates in pediatrics because of their composition. Compared to other NPs, the materials used to make them impart to NPs biocompatibility and biodegradability, non-immunogenicity and non-toxicity, and a high drug entrapment efficiency. Polymeric and lipid NPs are the most clinically approved NPs in therapy [18].

Other kinds of NPs show promising results, such as gold and silver NPs or quantum dots. Metallic NPs are promising for cancer diagnosis and for inhibiting tumor growth. For instance, Liu and co-workers [19] developed gold NPs coated with polyethylene glycol, chitosan and polyethyleneimine loaded with siRNA to reduce pediatric brain tumor resistance radiation therapy. Also, titanium nanohybrids were used to fabricate palatable pediatric formulation azithromycin, masking its bitter taste [20]. Despite good prospects as drug delivery systems, the use of metal NPs is compromised by their biosafety. Several studies have revealed that they can alter the cell cycle, reducing the cell survival rate [21,22].

The development of NPs for pediatric use must consider pediatric physiology [13]. NPs smaller than 5.5 nm are filtered by the kidney [23], whereas if they are between 200 and 500 nm they are filtered by the spleen, like adults; all NPs show an increased accumulation in children’s lungs regardless of the size, due to the airway diminished caliber and increased ventilation rate. Because the plasma protein level is lower in neonates and young infants, opsonization may be decreased. Pharmacokinetic parameters (circulation time, area under the curve and distribution) of NPs (100–200 nm) could be altered because children exhibit an increased cardiac output, a reduced glomerular filtration rate and an increased organ surface area to weight ratio [13]. Both the innate and adaptive immune systems are immature in newborns, and mature and acquire memory as they grow. Monocytes and macrophages are immature as well as neutrophil functions, and the efficiency of the adaptive immune system to respond to T-cell-dependent antigens early are impaired in neonates compared with older children and adults [24]. Therefore, the mononuclear phagocyte system (MPS) clearance of negatively charged particles larger than 20 nm may be decreased as well as the shape-dependent uptake; also, the serum protein binding of positively charged particles could be altered because the serum protein distribution is age-dependent [13]. The immaturity of cytochrome enzymes modifies the toxicity and degradation of NPs in pediatric patients, depending on their composition [13]. As regards the composition, poly(ethylene glycol) (PEG) is tolerated in children, and the lipid composition allows an increased half-life of the NPs, avoiding macrophage uptake; adults and children older than one year react in a similar manner [13]. The fenestration sizes of healthy and tumor tissues do not differ in adults and children, so the size of NPs could be considered for exploiting the enhanced permeability and retention (EPR) effect [25].

## 3. Polymeric Nanoparticles

**Figure 3:**
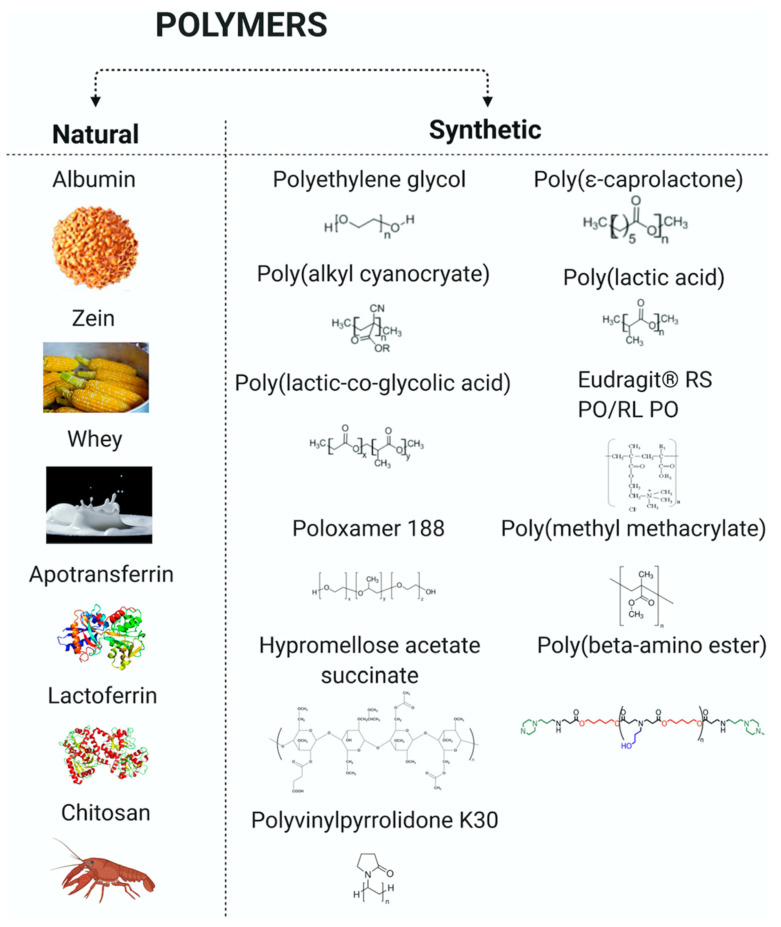
Natural and synthetic polymer used to make pediatric nanoparticles. It was created with Biorender (https://biorender.com/ accessed on 23 April 2021).

Polymeric NPs are a kind of solid NP whose matrix consists of natural, semi-synthetic or synthetic polymers. They can be biodegradable or non-biodegradable. Because non-biodegradable polymeric NPs can induce chronic toxicity and high immunological response after chronic use [26,27], biodegradable polymers are of great interest in pediatric nanomedicine. According to their origin, biodegradable polymers can be classified as natural and synthetic (Figure 3).

Synthetic polymers, like aliphatic polyesters, such as poly(lactic-co-glycolic acid) (PLGA), poly(glycolic acid) (PGA) and poly(lactic acid) (PLA), have been approved by the US Food and Drug Administration (FDA) as they are degraded as carbon dioxide and water by the human body [26,28]. Natural polymers can be proteins and polysaccharides. They are obtained from animal and vegetable sources. Examples of proteins are collagen, albumin, zein, gluten, whereas polysaccharides can be chitosan, hyaluronate, cellulose, alginate, starch, etc. NPs based on these polymers display great properties such as biodegradability and low toxicity due to their metabolism [29]. Polymeric NPs possess a high drug loading capacity [27] and can improve both pharmacodynamics and pharmacokinetics of formulated drugs, enhance safety, and obtain more available and suitable formulations in children [13].

### 3.1. Natural Polymeric Nanoparticles

Albumin is a water-soluble and biodegradable animal protein. It can be bovine and human serum albumin, and ovalbumin (from egg white). This protein improves the release of drug bioavailability and decreases drug resistance in the body [29]. It is non-toxic and non-immunogenic [30]. Albumin-bound technology (named nab-technology) was developed and used for the delivery of drugs in the treatment of various types of tumors [31].

Paclitaxel bound-albumin NPs (Abraxane^®^) have demonstrated efficacy in adults with solid tumors [32]. This formulation is the first approved product based on protein nanotechnology chemotherapeutics. Furthermore, Abraxane^®^ is the first natural polymeric nanoparticle used in pediatric patients. Abraxane^®^ is a lyophilized powder and is administered as a suspension in sodium chloride solution [33]. The diameter of paclitaxel bound-albumin NPs was 130 nm, which prevents any risk of capillary obstruction [33]. There is a clinical trial (ClinicalTrials.gov Identifier: NCT01962103) in phase I/II in pediatric patients with recurrent or refractory solid tumors, in phase I of which the maximum tolerated dose was determined. Compared to the conventional paclitaxel formulation containing Cremophor EL and ethanol as solvents, Abraxane^®^ has an improved safety profile [34]. Wagner and co-authors studied Abraxane^®^ for the treatment of pediatric bone sarcoma. These albumin NPs produced in vivo a temporary regression of an osteosarcoma model and increased survival [35].

Currently, a phase I clinical trial is being developed by Children’s Oncology Group based on albumin-bound rapamycin NPs administered by the intravenous route in association with irinotecan hydrochloride temozolomide, for the treatment of children with recurrent or refractory solid tumors (ClinicalTrials.gov Identifier: NCT02975882).

Catanzaro and collaborators developed redox-responsive bovine serum albumin NPs for the cisplatin release in medulloblastoma treatment. This brain tumor is one of the primary central nervous system cancers in the pediatric population. The desolvation method was applied to prepare them to use N, N’-bis(acryloyl)cystamine (BAC) as a crosslinker. BAC led to the release of cisplatin inside tumor cells where high glutathione levels were present. Spherical NPs showed a size range of 83 nm and a polydispersity index (PDI) of 0.3. Albumin nanocarriers were in vitro tested on Daoy medulloblastoma and healthy cells. Studies revealed a rapid and efficient internalization of the NPs and higher cytotoxicity on Daoy medulloblastoma cells than healthy controls. These redox-responsive bovine serum albumin NPs could improve the safety and efficacy of antineoplastic drugs in treating pediatric brain tumors [36].

NPs based on bovine serum albumin, hydroxyapatite, and paclitaxel were prepared for in situ treatment of osteosarcoma by Liu and collaborators. NPs were prepared by wet coprecipitation method, purified and freeze-dried; the particles obtained had 55 nm diameter, good drug loading (32.17%), sustained release properties of drug and calcium and low cytotoxicity. Moreover, NPs showed significant anticancer and inhibition of tumor metastasis activities, and osteogenesis effects [37].

Islam and co-workers studied safe and effective zein-whey NPs (ZWP) for the oral delivery of lopinavir and fenretinide in children. Zein is a biopolymer derived from corn cell endosperm. It is composed of glutamic acid and hydrophobic amino acids, leucine, proline and alanine [38]. Zein has been certified by the FDA as Generally Recognized As Safe (GRAS) polymer [29]. This corn protein is used as a preservative coating for some food and pharmaceuticals [39]. In nanotechnology, zein NPs show great potential in providing a controlled release of the drug. Due to their hydrophobicity, zein NPs can protect the encapsulated contents from the stomach acid [38]. Whey protein is a by-product of the manufacture of cheese or casein. This protein is interesting as a delivery material because of its GRAS status. It is composed of various globular proteins. Its main properties are the quality of binding lipophilic bioactive compounds and the ability to form hydrogel [40]. NPs were obtained by the phase separation method based on the different solubility of whey protein and zein in an aqueous solution and hydroalcoholic solvent. Zein formed the core and whey protein of the shell. The ZWP size ranged from 200 to 250 nm, and the encapsulation efficiency was over 70%. ZWP showed potent ex vivo and in vivo bioadhesive properties. In vivo, gastrointestinal distribution and pharmacokinetic studies were performed. As a result, ZWP had good sensory properties and sustained drug release; they improved the oral bioavailability of lopinavir and fenretinide and raised their half-life without showing any immunogenicity in mice [41].

Ahmed et al. proposed apotransferrin or lactoferrin NPs containing carboplatin to treat retinoblastoma in children representing a common pediatric malignant intraocular cancer. Apotransferrin and lactoferrin are iron transporting proteins characterized by high biocompatibility. Nanoformulations were prepared by the sol-oil method. Apotransferrin-carboplatin and lactoferrin-carboplatin nanoparticle sizes were 140 nm and 263 nm, and the PDI were 0.155 and 0.340, respectively. The encapsulation efficiency was 50% for Apo-transferrin and 52% for lactoferrin due to the electrostatic interaction of carboplatin with proteins. Both protein NPs were able to enter into the cells, probably by receptor mediated endocytosis, and showed in vitro sustained intracellular drug retention and, thus, anti-proliferative activity in retinoblastoma cells compared to the free drug [42]. These natural biodegradable proteins provide a formulation with a low toxicity profile for children.

Another natural polymer used for preparing pediatric NPs is chitosan, a chitin derivative characterized by biocompatibility and biodegradability [27]. Chen and collaborators proposed prednisolone-loaded chitosan NPs formulated in oral dispersible tablets for asthma treatment. Ionotropic external gelation method was used to prepare these NPs with sizes between 130 and 450 nm and a PDI of 0.310. The surfactant used was Tween^®^ 80, and the crosslinker was tripolyphosphate. The chitosan-tripolyphosphate weight ratios affected the encapsulation efficiency and prednisolone solubility most when a 1:1 ratio was employed. NPs were spherical and with a smooth surface [43]. The oral dispersible tablets obtained by direct compression of NPs could represent an efficient delivery system for pediatric populations who have swallowing problems.

### 3.2. Synthetic Polymeric Nanoparticles

As regards synthetic polymers, PEG is a biodegradable polymer often used in nanomedicine. The FDA approved it because of its biocompatibility and biodegradability [26]. Its hydrophilic nature does not accumulate in the tissues and can increase the drug solubility and stability in aqueous media [27].

Krishnan and co-authors developed NPs based on an amphiphilic block of the biodegradable copolymer, consisting of PEG and poly(ε-caprolactone) (PCL). The Nanoprecipitation method was used to prepare dexamethasone-loaded block copolymer NPs to treat leukemia in the pediatric population. This blood cancer is the most common in children and teens. The average particle size was 110 nm. In vitro cell studies and in vivo studies in mice from 4 to 8 weeks of age demonstrated no toxicity of unloaded nanoformulation. It was also possible to study dose reduction compared to the free drug, and it was observed a better quality of life and survival of mice [16].

Polymeric NPs of poly(isobutylcyanoacrylate) (PIBCA) based on the grafting of the anti-CD99 antibodies on the chitosan corona were developed by Ramon et al. The association of the antibodies on NPs’ surface can be achieved by designing targeted NPs with biotinylated ligands and using the biotin-streptavidin coupling method. Redox radical emulsion polymerization was used to synthesize PIBCA/chitosan NPs. The dimensions were less than 100 nm, which enables a better distribution on the body. These polymeric NPs release siRNA, which was acting on cell membrane glycoprotein CD99, and is overexpressed in these tumor cells. In vivo studies in mice revealed that the inhibition of Ewing sarcoma’s gene expression was 78%. The different polymers allowed siRNA, a polyanionic hydrophilic molecule, to bond to the cationic and hydrophilic shell of the nanoparticle chitosan corona resulting in a longer blood circulation time [44].

Yu and collaborators prepared salinomycin-loaded PEGylated PLGA nanoparticles (SAL-NP). SAL-NP conjugated with CD133 aptamers (Ap-SAL-NP) have the potential to deliver the drug and attack CD133+ osteosarcoma cancer stem cells. Osteosarcoma is another childhood bone cancer. The FDA has approved PLGA as a safe material, and it is often employed, such as in drug delivery systems improving circulation time and permeability [27]. NPs were prepared by emulsion solvent evaporation. Particle size was around 150 nm, and drug encapsulation efficiency was nearly 50%. In vitro and in vivo studies in mice 4–6 weeks of age showed that Ap-SAL-NP could selectively kill CD133+ osteosarcoma cancer stem cells [45].

Majumdar et al. developed a formulation of luteolin loaded in PLA-PEG NPs. The FDA already approves the polymers used. PLA is characterized by hydrophobicity, biodegradability, biocompatibility and a safe profile [27]. PLA is the core of the nanoparticle. Meanwhile, PEG is the hydrophilic shell and luteolin is located inside the inner core of the nanoparticle. Luteolin is a flavonoid that inhibits the activity of lung cancer cells and head and neck cancer ones; it has been investigated for pediatric use in chemoprevention (ClinicalTrials.gov Identifier: NCT03288298). This natural compound has low solubility in water, so it was incorporated in synthesized NPs. PLA-PEG NPs were prepared by the same method as mentioned above [46]; luteolin and the diblock copolymer PLA-PEG methanolic solution were added dropwise to 1% polyvinyl alcohol solution and, then, nanoparticle suspension was filtrated and purified. The diameter of the particles was about 115 nm. Anticancer activity was demonstrated in in vitro studies. In vivo studies in mice aged from 4 to 6 weeks showed that PLA-PEG NPs inhibited lung cancer cells’ growth and squamous cell carcinoma of head and neck cells compared with free luteolin [46].

Etoposide (VP-16) is a chemotherapy drug for glioma treatment; it is hydrophobic and has a poor brain parenchyma distribution. NPs of PLGA or PLGA/Poloxamer 188 loaded with VP-16 were developed by Callewaert and co-workers [47]. The particles were obtained by a modified spontaneous emulsification-solvent diffusion method; the size of NPs obtained was between 110 and130 nm and a PDI below 0.2. The results on two glioma cell lines showed an enhanced antitumor efficacy: in fact, PLGA and PLGA/Poloxamer 188 particles induce an improvement in the drug’s cytotoxicity activity.

Seremeta et al. studied micro-and NPs that consist of Eudragit^®^ RS PO and Eudragit^®^ RL PO loaded with benznidazole, a drug with low solubility, were studied to treat Chagas’ disease in neonates and young children. Eudragit^®^ RS PO polymers with low permeability and RL PO polymers with high permeability are copolymers of acrylic and methacrylic acid esters. Spray-drying was used to prepare microparticles, while NPs were prepared by nanoprecipitation and freeze-drying. Pluronic^®^ F68 was used as a steric stabilizer in NPs and was suitable to keep the optimal particle size. The nanoparticle size was between 200 and 300 nm and a PDI below 0.2, whereas microparticles were below 2 μm. The in vitro dissolution rate of benznidazole was increased, and the nanocarriers’ release was due to the diffusion from the polymer matrix [48].

Deng and co-workers recently developed lopinavir and ritonavir NPs based on Eudragit^®^ E PO as new orodispersible drug delivery systems. NPs were obtained by nanoprecipitation method using methanol for dissolving drugs and polymer and a Kolliphor^®^ P 188 aqueous solution. After the removal of the organic solvent, nanosystems were purified and freeze-dried. The powders were rapidly dispersible in water (in about 7 s) with a particle size of 158.2 ± 1.1 nm and 125.2 ± 3.7 nm for lopinavir and ritonavir NPs, respectively. NPs were able to mask the unpleasant taste, and improve the dissolution and oral bioavailability of drugs compared to commercial tablets (Kaletra^®^) and raw drugs [49].

Zaritski and collaborators studied galactomannan-based NPs for obtaining selective intratumoral accumulation in pediatric sarcomas. Galactomannan was hydrolyzed and grafted with poly(methyl methacrylate) using a 2:1 weight ratio. Galactomannan is a biocompatible and biodegradable polysaccharide, while poly(methyl methacrylate) is a biocompatible and non-biodegradable polymer. NPs of 141 nm in size and a PDI of 0.11 were selected for in vitro and in vivo studies due to their suitable size. The encapsulation capacity was demonstrated with imatinib. NPs were internalized in most of the cells that overexpress actively target glucose transporter-1 (GLUT-1). This intratumoral accumulation was also confirmed in pediatric patient-derived models of solid tumors [50].

Monterrubio et al. developed polymeric NPs conjugated to anti-GD2 antibodies and loaded with topoisomerase-I inhibitor SN-38 to treat pediatric neuroblastoma. PLGA and carboxymethyl-poly(ethylene glycol)-b-poly(lactic acid) (PLA-PEG-COOH) were chosen as polymers. The surfactant used was polyvinyl alcohol. NPs were obtained by a single oil-in-water emulsion method. The mean size of particles was about 300 nm and a PDI below 0.3, and zeta potential of about −22 mV. High loading efficiency was obtained and an in vitro controlled drug release (48–72 h). The in vivo tumor penetration of the drug was increased in mice when released from targeted NPs compared with non-targeted ones or free SN-38, prolonging mice’s survival [51].

Clofazimine NPs based on hypromellose acetate succinate (HPMCAS), lecithin and zein were studied by Zhang and co-authors. This poorly soluble drug is used in treating Cryptosporidiosis, a disease that can be lethal in children. HPMCAS enhances the drug solubility and, in conjunction with lecithin and zein, provides stability to the NPs. These NPs were prepared by the flash nanoprecipitation process and dried by freeze-drying or spray-drying to produce redispersible powders. HPMCAS NPs showed the greatest particle size stability. Particles with sizes in the range of 70–100 nm, PDI of 0.19–0.26 and high encapsulation efficiency of over 92% were obtained. This formulation improved the dissolution rate of clofazimine and the supersaturation levels compared to the marketed formulation and, thus, was proposed as a pediatric fast-releasing oral formulation [52].

An oral nanosuspension for mefenamic acid delivery was investigated by Perween and collaborators. To improve its poor water solubility, mefenamic acid was incorporated in NPs composed of hydroxypropyl methylcellulose and Tween 80 or sodium dodecyl sulfate as surfactants. Formulations were prepared by antisolvent precipitation method. NPs, with a size diameter of 510 nm and a PDI of 0.329, were stable for more than three weeks; the drug content was 84.41%. In vitro release study demonstrated that the drug release increased when high surfactant concentration was used; a reduction in the particle size was also obtained [53]. Further studies are necessary to explore their complete potential for pediatric delivery.

Uckun et al. characterized multi-functional NPs as a novel nanoformulation anti-acute lymphoblastic leukemia. CD22-RTM (RNA trans-splicing molecules) was complexed with PVBLG-8, a cationic polypeptide, in a weight ratio of 1:10. This polypeptide displayed safety, biocompatibility, pharmacodynamic and biodistribution profiles. It was suitable for nucleic acid delivery in vivo due to its ability to enhance membrane permeability. The particle size was 105 nm and a PDI of 0.192. This nanosystem revealed anti-leukemia activity in vitro [54].

A cancer gene therapy based on poly(beta-amino ester) (PBAE) NPs was studied by Choi and collaborators to treat brain tumors. PBAE was self-assembled with plasmid DNA encoding the suicide gene herpes simplex virus I thymidine kinase at a 1:1 volume ratio through transfection. The size range was 100–200 nm. These polymers are an interesting biomaterial for non-viral gene delivery due to their fast degrading under cytotoxicity. In vivo studies revealed an increased survival compared to previous studies, so it is a promising pediatric nanomedicine [55].

González et al. developed a dry nanosuspension of praziquantel, using Poloxamer 188 and Polyvinylpyrrolidone K30 as stabilizers, to treat schistosomiasis. Preschool-age children are involved in the transmission of this parasitic infection. A marketed formulation is a tablet form, and it is not suitable for pediatric patients. Praziquantel is characterized by its low aqueous solubility and a solidly bitter and metal taste. High-pressure homogenization was used to prepare NPs that were dried by spray-drying and then redispersed in a sweet vehicle. This system improves the aqueous solubility and dissolution performance of praziquantel [56].

The polymeric NPs studied for the treatment of pediatric disease are displayed in Table 1.

## 4. Lipid Nanoparticles

Lipid-based NPs form colloidal dispersions. These drug delivery systems can be emulsions, vesicular systems and lipid particulate systems [57]. Liposomes are vesicles having one or more phospholipid bilayers. Phospholipids consist of a hydrophilic head and a hydrophobic tail: the apolar chains face each other, whereas the polar heads are oriented towards the aqueous medium and in the central compartment forming the liposome. The hydrophilic drugs occupy the aqueous compartment; meanwhile, the lipophilic ones are attached to the liposomal lipid membrane bilayer by the hydrophobic interaction [57]. Liposomes range in size between 20 nm and 10 μm [58]. Solid lipid nanoparticles (SLNs) were identified as a viable alternative to various colloidal systems. SLNs have a spherical shape with a dimension ranging from 50 nm to 1000 nm, and they are composed of a solid lipid core stabilized in the aqueous medium by surfactants [57]. They present high biocompatibility because lipids are well tolerated by the body and have reduced cellular and systemic toxicity. SLNs improve drug stability due to the drug’s protection from chemical and enzymatic degradation, and enhance its bioavailability [59]. Due to the high lipid biocompatibility, they can be administered through all existing routes of administration. Since they consist of hardcore solid lipids at room temperature, SLNs can guarantee stability and controlled drug release and improve the drug delivery to its target.

Nanostructured lipid carriers (NLCs) differ from SLNs because their core is made up of both solid and liquid lipids. NLCs show higher drug loading capacity than SLNs because their nucleus is less organized [57,60].

Lipid drug delivery nanosystems are good candidates to encapsulate poorly water-soluble, lipophilic drugs and poorly permeable drugs [60]; particular attention should be paid to the choice and concentration of lipids because, as mentioned above, they can reduce the clearance and increase the plasma half-life of the formulation [13,60].

Lipid-based NPs studied for the treatment of pediatric diseases are summarized in Table 2.

### 4.1. Liposomes

Several liposomes are in the market and have been evaluated in children (Table 3). They were developed for use in the adult population, but they have been used in clinical trials in the pediatric population. The route of administration is intravenous, and most of them are used in anticancer therapy.

Vincristine sulphate liposome injection (marketed as Marqibo^®^) is a formulation used in treating lymphoblastic leukemia. Marqibo^®^ liposome is composed of sphingomyelin and cholesterol in approximately 60:40 molar ratio with a particle size around 100 nm [61]. It was approved in adults by the FDA in 2012, and a phase I clinical trial was reported utilizing Marqibo^®^ (ClinicalTrials.gov Identifier: NTC02879643) in pediatric patients aged <21 years with relapsed or chemotherapy-refractory solid tumors or leukemia [62]. The study did not show dose-limiting neurotoxicity with the adult FDA-approved dose. Nonclinical studies demonstrated that these liposomes could increase the residential time, accumulation in the tumor tissue and controlled release of vincristine in the tumor compared with conventional vincristine [63].

Intrathecal liposomal cytarabine (marketed as DepoCyt^®^ in 1999) has been approved in children with leptomeningeal dissemination [25]. Currently, a clinical trial in phase 2 (ClinicalTrials.gov Identifier NCT02393157) by New York Medical College is in progress. Depocyt^®^ is administered intrathecally in children, adolescents and young adults with relapsed CD20 positive B-cell Non-Hodgkin Lymphoma. This multivesicular liposome platform composed of cholesterol, triolein, dioleoil phosphatidylcholine and dipalmitoyl phosphatidyl glycerol allowed a more extended drug-controlled release with higher exposure than standard cytarabine, avoiding frequent multiple injections of this drug characterized by its short half-life into the cerebrospinal fluid [61]. Pharmacokinetics and toxicity were evaluated in children and adolescents with malignant brain tumors because the variation in intracranial volume with age is a difficulty in dosage in the pediatric population. The doses studied exhibited sufficient drug exposure for at least one week and appeared well tolerated [64].

In 1996, daunorubicin (DaunoXome^®^) was approved by the FDA to treat advanced human immunodeficiency virus (HIV)-related Kaposi’s sarcoma in adults. A phase III clinical trial of liposomal daunorubicin added to granulocyte colony-stimulating factor showed a good result for pediatric relapsed acute myeloid leukaemia [65]. Studies suggest that liposomal formulation is effective and less cardiotoxic in pediatric patients. Daunorubicin was encapsulated into small unilamellar liposomes with a range of 45 nm, composed of highly purified distearoyl phosphatidylcholine and cholesterol, in a concentration of 2:1 mole ratio, respectively [61]. The liposome structure enhances the stability and increases the half-life of DaunoXome^®^ compared with free Daunorubicin [61].

Doxil^®^ (approved 1995), the first nanomedicine available on the market, is pegylated liposomal doxorubicin (90 nm) consisting of hydrogenated soybean phosphatidylcholine, cholesterol and N-(carbonyl-methoxypolyethyleneglycol2000)-1,2-distearoyl-sn-glycero-3-phosphoethanolamine sodium (56:39:5 molar ratio), whereas Myocet^®^ (marketed in 2000) is non-pegylated liposomal doxorubicin (100–200 nm) consisting of phosphatidylcholine and cholesterol (55:45 mole per cent) [66]. Both liposomes have been studied in clinical trials in pediatric patients. Doxil^®^ was combined with other drugs in a clinical trial (ClinicalTrials.gov Identifier: NCT00006029) to treat Hodgkin lymphoma. The results have shown a well-tolerated regimen for relapsed Hodgkin lymphoma with similar results to more toxic regimens [67]. However, Myocet^®^ with other drugs have been evaluated in patients cardiopathic affected by non-Hodgkin lymphoma (ClinicalTrials.gov Identifier: NCT01009970). The results are not yet publicly available. These two formulations have different pharmacokinetics and toxicity profiles, but doxorubicin free liposomal reduces this drug’s cardiotoxicity characteristics [68]. Doxil^®^ pharmacokinetics was evaluated in pediatric patients. The studies demonstrated differences compared to adults; a low half-life may be associated with decreased protein binding of the drug due to reduced plasma protein concentrations in the pediatric population [13].

Mepact^®^ is a liposomal formulation containing mifamurtide, a synthetic analogue of a cell wall component of Mycobacterium species that stimulates the immune system to destroy the tumor cells. Mepact^®^ is composed of multilamellar liposomal formulation under 100 nm, based on synthetic lipids such as 1,2-dioleoyl-sn-glycero-phosphoserine and palmitoyl-2-oleoyl-sn-glycero-3-phosphocoline (3:7 molar ratio) [61]. Mepact^®^ is indicated for treating high-grade resectable non-metastatic osteosarcoma after macroscopically complete surgical resection in children, adolescents and young adults; the market authorization was in 2009 as a second-line treatment for children [69].

In 2017, Vyxeos^®^ or CPX35 was approved for recurrent leukemia in adults [70]. This lipid formulation is the first liposome loaded with both Daunorubicin and Cytarabine. This medicine contains bilamellar liposome composed of 1,2-Distearoyl-sn-glycero-3-phosphocholine, 1,2-distearoyl-sn-glycero-3-phospho-(1-rac-glycerol), and cholesterol corresponds to the molar ratio 7:2:1 and with a diameter of 100 nm [70]. A phase I/II clinical trial (ClinicalTrials.gov Identifier: NCT02642965) reports that Vyxeos^®^ can reduce the heart’s side effects and studies the best dose liposome-encapsulated in a pediatric population from 1 to 21 years. Currently, a phase I pilot study (ClinicalTrials.gov Identifier: NCT03826992) is testing the safety and tolerability of venetoclax combined with Vyxeos^®^. The liposomal formulation exerts a synergistic effect on leukemia cells compared with free drugs that shows a different pharmacokinetics profile. An enhancement in overall survival has been observed in adult patients treated with the liposomal formulation compared to those tested with the free drug [70]. In the future, these data could be extrapolated to the pediatric population.

Liposomal Amphotericin B (marketed as AmBisome^®^) is used in treating systemic fungal infections, approved in 1997 in adults [61]. AmBisome^®^ are small unilamellar liposomes of 100 nm made up of hydrogenated soy phosphatidylcholine, cholesterol and distearoyl phosphatidylglycerol (2:1:0.8 molar ratio) [61,71]. The lipidic complex interacts with Amphotericin B providing stability to the formulation, reducing toxicity and achieving a prolonged blood residence time [71]. Immunocompromised children and adolescents were admitted to a study for assessing the safety, tolerability and pharmacokinetics of AmBisome^®^. The results show that a similar liposomal formulation dose can be used in adult and pediatric patients, but the latter can develop azotemia [72].

### 4.2. Solid Lipid Nanoparticles and Nanostructured Lipid Carriers

González-Fernández et al. proposed different oral lipid NPs to deliver anti-osteosarcoma drugs [73]. Firstly, they prepared lipid NPs encapsulating edelfosine or methotrexate and evaluated the in vitro activity in various primary and commercial osteosarcoma cell lines. Edelfosine-based lipid NPs were obtained using Precirol^®^ ATO 5 and Tween^®^ 80 by hot homogenization method followed by high shear homogenization and ultrasonication. The size and the zeta potential of the obtained particles is about 137 nm and −20 mV, respectively. Osteosarcoma cell studies demonstrated that the encapsulation of edelfosine in lipid NPs increased the antitumor efficacy due to the higher uptake in primary and metastatic cells [73]. The same group prepared doxorubicin lipid NPs and studied the antitumor efficacy alone and combined with edelfosine lipid NPs. Doxorubicin lipid NPs were prepared by three different methods (O/W emulsion, W/O/W emulsion both followed by solvent evaporation and hot melting homogenization), but only the hot melting homogenization allowed them to obtain small homogeneous particles (95 nm size and 0.2 PDI) and high drug loading [74]. The lipid phase was constituted of Precirol^®^ ATO 5, triethanolamine, oleic acid and doxorubicin; the aqueous phase contained Tween^®^ 80 and EDTA. This nanoformulation was able to counteract neoplastic cells’ drug resistance by increasing cellular absorption and reducing doxorubicin’s efflux. Moreover, a synergistic effect of doxorubicin and edelfosine lipid NPs was demonstrated [74].

A pediatric oral liquid formulation of hydrochlorothiazide for pediatric hypertension was investigated by Cirri et al. It is a drug characterized by low solubility and poor permeability. Drug–cyclodextrin complex was loaded in solid lipid NPs. SLNs were obtained by a hot high-shear homogenization method followed by ultrasonication. The leader formulation was prepared with Precirol^®^ ATO5 as lipid and Pluronic^®^F78 as surfactant. SLNs revealed a size of 380 nm, and hydroxylpropyl-beta-cyclodextrin improved NPs stability. This nanoformulation was tested on Sprague–Dawley rats showing a controlled drug delivery and improved oral bioavailability compared with drug suspension [75]. Afterwards, they studied nanostructured lipid carriers (NLC) instead of SLNs. The microemulsion technique was chosen to produce hydrochlorothiazide lipid NPs with a 300–400 nm range size. The NLC that demonstrated better results consisted of Precirol^®^ ATO5 as solid lipid, Tween^®^ 80 and Tween^®^ 20 as a surfactant and co-surfactant, and castor oil as liquid lipid. Formulation orally administered in Sprague–Dawley rats showed an enhanced therapeutic effect. Moreover, this NLC showed better sustained drug release and increased diuretic effect than SLNs [76]. Starting from these results, the same authors recently published a new formulative study regarding SLN and NLC as carriers of hydrochlorothiazide based on Precirol^®^ ATO5 and Transcutol^®^ HP as solid and liquid lipids, respectively, and Gelucire^®^ 44/14, PluronicF68 or Tween^®^ 80 as surfactants. As demonstrated previously, NLC showed a better loading capacity and drug release rate than SLN; in particular, Gelucire^®^ 44/14 improved particle size, entrapment efficiency, drug release properties and storage stability of new SLN and NLC and also concerning previously developed formulations [75,76]. Due to their non-toxicity and the ability to pass the Caco-2 cell monolayer, these formulations appear suitable for developing oral liquid pediatric formulations [77].

### 4.3. Other Type of Lipid Nanoparticles

In situ self-assembly nanoparticles (ISNPs) were also proposed. Lopinavir and ritonavir loaded in self-assembly nanocapsules were studied by Pham and collaborators. They developed solid palatable granules based on oleic acid and α-tocopheryl polyethylene glycol 1000 succinate (1:2 ratio) plus colloidal silicon dioxide (Aeropearl^®^ 300); when in contact with water or biological fluids, oleic acid and α-tocopheryl polyethylene glycol 1000 succinate (1:2 ratio) self-assemble to form ISNPs encapsulating both drugs. The size of ISNPs obtained was 158 nm. When orally administered in rats, improved lopinavir bioavailability and concentration in the brain and lymphoid tissues, sanctuary sites of HIV, were observed [78].

Rodríguez-Nogales and co-workers studied a suitable candidate for childhood cancer therapy. They prepared and characterized nanoassemblies using squalenoyl-gemcitabine and alkyl-lysophospholipid edelfosine by nanoprecipitation method. The nanoassemblies with a mean size of 50 nm showed high uptake in human osteosarcoma cells and an excellent antitumor profile. In vivo studies were carried out in 6–8 weeks old mice and revealed an enhancement in gemcitabine and edelfosine pharmacokinetic profiles [79].

## 5. Considerations and Challenges in the Application of Polymeric and Lipid Nanoparticles in Pediatrics

The literature analysis showed that polymeric and lipid NPs have been widely studied to treat pediatric diseases. In particular, albumin NPs and liposomes are also used in clinical practice or are the object of clinical trials conducted with the pediatric population.

Several final considerations can be drawn. Firstly, all prepared formulations use excipients approved as food ingredients generally recognized as safe (GRAS) or as an inactive ingredient for drug products. Knowledge of the excipient safety profile is one of the regulatory agencies’ mandatory requirements for avoiding any potentially toxic or unsuitable excipients for children. Moreover, the preparation methods reported were also techniques that limit the use of organic solvents, mainly chlorinated ones. Concerning the size of NPs, most of the particles proposed/used have a mean diameter of 150 nm, mainly those intravenously administered, to avoid capillary obstruction and increase the loaded drug’s pharmacokinetics. Secondly, all nanoformulations prepared are almost exclusively focused on cancer treatment that is a challenge both for the adult and pediatric population. Only a few NPs are intended to treat pediatric chronic diseases (e.g., hypertension, asthma). Therefore, nanomedicine applied in pediatrics remains a significant challenge in the near future.

NPs could indeed solve many of pediatric medicines’ needs, such as developing age-appropriate formulations for each pediatric group, due to their ability: (i) to modify the physicochemical and pharmacokinetic properties of loaded drugs; (ii) to mask the unpleasant taste of drugs, thus improving the palatability; (iii) to be easily swallowable even for newborns; (iv) to achieve a prolonged drug release and, thus, the reduction of the number of doses and the enhancement of compliance both of patient and parents; (v) in drug targeting; (vi) to facilitate easy dose adjustments as children grow; (vii) and enable combination of more drugs in one formulation (Drug Combination Nanoparticle Platform). Many of these advantages have been highlighted in a recent report, about innovative delivery systems for pediatric medicine, by the World Health Organization and Unitaid, which reports the NPs as a technology that may be applied to more than one dosage form type and/or route of administration, and which may have relevance for pediatric product development [80]. Moreover, foundations, such as The Center for Pediatric Nanotechnology of Emory University School of Medicine and Children’s Healthcare of Atlanta, promote the development of pediatric nanomedicine focus in pediatric heart disease, thrombosis and infectious diseases [81].

The promising results of in vitro and ex vivo studies obtained with NPs, reported here, are certainly an encouraging basis for further research and their application in clinical practice. However, the unavailable data on pharmacokinetics, dose, safety and efficacy of many drugs in pediatric patients limits the development of NP formulations. The need for clinical trials on the pediatric population was widely recognized and is stimulated by EU legislation. Currently, 1,534 trials with a EudraCT protocol are reported in the EU Clinical Trials Register database; an increase in the next years is desirable (https://www.clinicaltrialsregister.eu/; accessed on 26 April 2021).

For the effective use of NPs in pediatrics, an increase in in vivo studies on nanoparticles’ efficacy and safety is necessary. In addition to the trials listed above, the list of recent clinical trials is scarce. Looking at the trials reported in the EU Clinical Trials Register database, only 31 trials concern nanoparticles and only 2 trials involve pediatric patients: one is related to the safety, tolerability, potential efficacy, and dose finding of INP20, an oral NPs for treatment of immunotherapy in peanut-allergic patients (EudraCT Number: 2018-003665-34); and the other the immunogenicity and safety of a respiratory Syncytial Virus (RSV) nanoparticle vaccine with aluminum in pregnant women and their infants. In the US ClinicalTrials.gov, three new clinical trials are also reported concerning the clinical assessment of oxiconazole SLNs in gel against tinea fungal infection (ClinicalTrials.gov Identifier: NCT03823040), the in vivo susceptibility of root canal bacteria to chitosan NPs (ClinicalTrials.gov Identifier: NCT03588351), and the side effects and best dose of albumin-bound rapamycin NPs when given together with temozolomide and irinotecan hydrochloride in treating pediatric patients with solid tumors (ClinicalTrials.gov Identifier: NCT02975882). The breakthrough of nanotechnology in pediatrics is certainly associated also with nanomedicine development in adults, and adults’ physiologically based pharmacokinetic (PBPK) models continue to prevail over pediatric models.

Finally, the development of new drug delivery for the pediatric population is strongly related to the progress of in vitro models simulating characteristics of the targeted pediatric population, with the purpose of facilitating in vitro–in vivo correlations (IVIVC).

## 6. Conclusions

Polymeric and lipid NPs have been widely studied to treat pediatric diseases, but only a few are used in therapy and only to treat pediatric cancers. Great attention is paid to the excipients used, to the safe and easy-to-scale-up preparation methods and, of course, to the size in order to obtain safe carriers.

This state of the art demonstrates that NPs are able to modify the physicochemical and pharmacokinetic properties, mask the unpleasant taste, obtain a prolonged or accelerated release and increase the efficacy of loaded drugs. Therefore, NPs could be favorable when developing new pediatric medicines not just to treat cancer but also for chronic disease, avoiding off-label use and extemporaneous formulations. Furthermore, NPs can be used as platforms for developing age-appropriate formulation for each pediatric group as required by the EMA and FDA.

The development of in vitro models, to facilitate IVIVC, as well as the increase of in vivo studies on pharmacokinetics, safety and efficacy of both drugs and nanoparticles, in pediatric patients, are necessary for guiding the research towards new pediatric drug delivery nanosystems.

## Figures and Tables

**Table 1:** Polymeric nanoparticles studied as drug carriers in pediatrics.

Drug	Composition	Pediatric Use/Indication	Reference
Paclitaxel	Albumin	Recurrent or refractory solid tumours Bone Sarcoma	[31,32,33,34] [35]
Cisplatin	Bovine serum albumin and BAC	Medulloblastoma	[36]
Paclitaxel	Bovine serum albumin and hydroxyapatite	Osteosarcoma	[37]
Lopinavir/Fenretinide	Zein and whey protein	Human immunodeficiency virus (HIV)	[41]
Carboplatin	Apo-transferrin and lactoferrin	Retinoblastoma	[42]
Prednisolone	Chitosan, Tween^®^ 80 and tripolyphosphate	Asthma	[43]
Dexamethasone	Poly(ethylene glycol) and poly (ε-caprolactone)	Leukaemia	[16]
siRNA	Poly(isobutylcyanoacrylate) and chitosan	Ewing sarcoma’s	[44]
Salinomycin	PEG-Poly(lactic-co-glycolic acid) and CD133 aptamers	Osteosarcoma	[45]
Luteolin	Poly(lactic acid), PEG and polyvinyl alcohol	Carcinoma of head and neck	[46]
Etoposide (VP-16)	Poly(lactide-co- glycolide) and poloxamer 188	Glioma	[47]
Benznidazole	Eudragit^®^RS PO, Eudragit^®^RL PO and Pluronic^®^F68	Chagas	[48]
Lopinavir/Ritonavir	Eudragit^®^ E PO and Kolliphor^®^ P 188	Human immunodeficiency virus (HIV)	[49]
Imatinib	Hydrolyzed galactomannan and poly(methyl methacrylate)	Sarcoma	[50]
SN-38 (irinotecan analog)	Poly(lactic-co-glycolic acid), carboxymethyl-poly(ethylene glycol)-b-poly(lactic acid) and polyvinyl alcohol	Neuroblastoma	[51]
Clofazimine	Hypromellose acetate succinate, lecithin and zein	Cryptosporidiosis	[52]
Mefenamic acid	Hydroxypropyl methylcellulose and Tween^®^ 80 or sodium dodecyl sulfate	Antipyretic	[53]
CD22-RTM	PVBLG-8 cationic polypeptide	Acute lymphoblastic leukaemia	[54]
Herpes simplex virus I thymidine kinase (HSVtk)	Poly(beta-amino ester)	Brain tumours	[55]
Praziquantel	Polyvinilpirrolydone K30 and polaxamer 188	Schistosomiasis	[56]

**Table 2:** Lipid nanoparticles studied as drug carriers in pediatrics.

Drug	Composition	Pediatric Use/Indication	Reference
Vincristine sulfate	Liposome based on Sphingomyelin:Cholesterol (60:40)	Lymphoblastic leukaemia	[61,62,63]
Cytarabin	Liposome based on Cholesterol:Triolein:Dioleoil phosphatidylcholine: Dipalmitoyl phosphatidyl glycerol (11:1:7:1)	Leptomeningeal dissemination	[25,61,64]
Daunorubicin	Liposome based on Distearoyl phosphatidylcholine: Cholesterol (2:1)	Acute myeloid leukaemia	[61,65]
Doxorubicin	Pegylated liposomal based on Hydrogenated soybean phosphatidylcholine:Cholesterol:PEG 200-DSPE (56:39:5)	Hodgkin lymphoma	[66,67,68]
Doxorubicin	Non-pegylated liposomal composed by Phosphatidylcholine:Cholesterol (55:45)	Non-Hodgkin lymphoma	[66,68]
Mifamurtide	Liposome based on Dioleoyl-sn-glycero-phosphoserine:Palmitoyl-2-oleoyl-sn-glycero-3-phosphocholine (3:7)	Osteosarcoma	[61,69]
Daunorubicin/Cytarabine	Liposome composed by 1,2-Distearoyl-sn-glycero-3-phosphocholine:1,2-distearoyl-sn-glycero-3-phospho- (1-rac-glycerol):Cholesterol (7:2:1)	Acute myeloid leukaemia	[70]
Amphotericin B	Liposome based on Hydrogenated soy phosphatidylcholine: Cholesterol: Distearoyl phosphatidylglycerol (2:1:0.8)	Systemic fungal infections	[61,71,72]
Edelfosine/methotrexate	Lipid Nanoparticle based on Precirol^®^ ATO 5 and Tween^®^ 80	Osteosarcoma	[73]
Doxorubicin	Precirol^®^ ATO 5, triethanolamine, oleic acid, Tween^®^ 80 and EDTA	Osteosarcoma	[74]
Hydrochlorothiazide	Solid lipid Nanoparticle based on hydroxylpropyl-beta-cyclodextrin, Precirol^®^ ATO5 and Pluronic^®^ F78	Hypertension	[75]
Hydrochlorothiazide	Nanostructured Lipid Carrier based on Precirol^®^ ATO5, Tween^®^ 80, Tween^®^ 20 and castor oil	Hypertension	[76]
Hydrochlorothiazide	Solid lipid nanoparticle and nanostructure lipid carrier based on Precirol^®^ ATO5, Transcutol^®^ HP, Gelucire^®^ 44/14 and Pluronic F68 or Tween^®^ 80	Hypertension	[77]
Lopinavir/Ritonavir	Nanocapsule containing Oleic acid and-α- tocopheryl polyethylene glycol 1000 succinate and Aeropearl^®^ 300	Human immunodeficiency virus (HIV)	[78]
Gemcitabine/Edelfosine	Nanoassembly based on squalenic acid and ether lipid	Osteosarcoma and neuroblastoma	[79]

**Table 3:** Nanoparticle formulations in the market.

Brand Name	Drug	Approval	Composition	Pediatric Use/Indication	Reference
Abraxane^®^	Paclitaxel	2005	Natural polymer: albumin	Solid tumours	[31,32,33,34]
Marqibo^®^	Vincristine sulfate	2012	Sphingomyelin:Cholesterol (60:40)	Lymphoblastic leukaemia	[61,62,63]
DepoCyte^®^	Cytarabin	1999	Cholesterol:Triolein:Dioleoil phosphatidylcholine: Dipalmitoyl phosphatidyl glycerol (11:1:7:1)	Leptomeningeal dissemination	[25,61,64]
DaunoXome^®^	Daunorubicin	1996	Distearoyl phosphatidylcholine: Cholesterol(2:1)	Acute myeloid leukaemia	[61,65]
Doxil^®^	Doxorubicin	1995	Hydrogenated soybean phosphatidylcholine:Cholesterol:PEG 200-DSPE (56:39:5)	Hodgkin lymphoma	[66,67,68]
Myocet^®^	Doxorubicin	2000	Phosphatidylcholine: Cholesterol (55:45)	Non-Hodgkin lymphoma	[66,68]
Mepact^®^	Mifamurtide	2009	Dioleoyl-sn-glycero-phosphoserine:Palmitoyl-2-oleoyl-sn-glycero-3-phosphocholine(3:7)	Osteosarcoma	[61,69]
Vyxeos^®^o CPX35	Daunorubicin/Cytarabine	2017	1,2-Distearoyl-sn-glycero-3-phosphocholine:1,2-distearoyl-sn-glycero-3-phospho- (1-rac-glycerol):Cholesterol (7:2:1)	Acute myeloid leukaemia	[70]
AmBisome^®^	Amphotericin B	1997	Hydrogenated soy phosphatidylcholine: Cholesterol: Distearoyl phosphatidylglycerol (2:1:0.8)	Systemic fungal infections	[61,71,72]

## Data Availability

Not applicable.
